# Spatial weight matrix in dimensionality reduction reconstruction for micro-electromechanical system-based photoacoustic microscopy

**DOI:** 10.1186/s42492-020-00058-6

**Published:** 2020-09-30

**Authors:** Yuanzheng Ma, Chang Lu, Kedi Xiong, Wuyu Zhang, Sihua Yang

**Affiliations:** 1 MOE Key Laboratory of Laser Life Science & Institute of Laser Life Science, College of Biophotonics, South China Normal University, Guangzhou, 510631 China; 2 Guangdong Provincial Key Laboratory of Laser Life Science, College of Biophotonics, South China Normal University, Guangzhou, 510631 China

**Keywords:** Photoacoustic microscopy, Spatial weight matrix, Dimensionality reduction, Distortion correction, Mutual information

## Abstract

A micro-electromechanical system (MEMS) scanning mirror accelerates the raster scanning of optical-resolution photoacoustic microscopy (OR-PAM). However, the nonlinear tilt angular-voltage characteristic of a MEMS mirror introduces distortion into the maximum back-projection image. Moreover, the size of the airy disk, ultrasonic sensor properties, and thermal effects decrease the resolution. Thus, in this study, we proposed a spatial weight matrix (SWM) with a dimensionality reduction for image reconstruction. The three-layer SWM contains the invariable information of the system, which includes a spatial dependent distortion correction and 3D deconvolution. We employed an ordinal-valued Markov random field and the Harris Stephen algorithm, as well as a modified delay-and-sum method during a time reversal. The results from the experiments and a quantitative analysis demonstrate that images can be effectively reconstructed using an SWM; this is also true for severely distorted images. The index of the mutual information between the reference images and registered images was 70.33 times higher than the initial index, on average. Moreover, the peak signal-to-noise ratio was increased by 17.08% after 3D deconvolution. This accomplishment offers a practical approach to image reconstruction and a promising method to achieve a real-time distortion correction for MEMS-based OR-PAM.

## Introduction

Photoacoustic microscopy (PAM) is a hybrid imaging technique, which benefits from the effect of laser-induced ultrasound in the designed optical absorbers [1–3]. However, most of the signals acquired in OR-PAM interfere with the superposition of the surrounding signals. Thus, the system has a high entropy, and thoroughly decoding the detected signal is a challenge [4, 5]. One of the methods to address this challenge is to simplify the acquisition system and construct a straightforward linear back-projection algorithm. Another effective approach is to improve the processing algorithm to decode a larger amount of information [6, 7].

When simplifying the acquisition system, micro-electromechanical system (MEMS)-based optical-resolution photoacoustic microscopy (MOR-PAM) provides a quasi-pointwise detection with straightforward structures. A MEMS mirror has the advantages of high-speed scanning, minimum power consumption, a comparatively small size, and light weight. Thus, it is suitable for a variety of OR-PAM instruments in rapid microscopic imaging [8], including a high-speed dual-view photoacoustic imaging pen [9], a MEMS scanning mirror enhanced photoacoustic laparoscope with an adaptive resampling method [10], and a combination of a MEMS scanning mirror and multimode fibers [11]. A MEMS mirror is selected for OR-PAM based on its intended application. An electrothermal MEMS has a relatively high driving force and a large field of view (FOV) than other types of MEMS. By contrast, an electrostatic MEMS has an accessible linearized-scanning mode [12]. The inevitable nonlinear response of a MEMS under the tilt angular-voltage curve, particularly at the end of each B-scan, leads to a distortion in the maximum amplitude projection (MAP) images. This distortion is particularly observed when imaging using a thermoelectric MEMS [13, 14], and the MEMS mirror is suitably calibrated before use for microscopy. The step size for a spot controlled using a MEMS mirror is smaller than the spot size, and the receiving area of the ultrasonic sensor leads to an interaction between the signal and ultrasonic sensor, which lasts for a certain period of time. Thus, a deconvolution is required for depth-coded microscopy with a small detection area.

One approach to improving the processing algorithm to decode a larger amount of information is to trade-off between the accuracy and time consumption during the engineering phase. Based on this idea, many studies on OR-PAM have been widely conducted in recent years to combine algorithms with different systems. In Hamid Moradi’s work, a deconvolution-based inversion with a sparsity regularization is proposed to improve the photoacoustic resolution [15], and the work of Heinz Roitner, a deconvolution was applied to provide a partial amplitude compensation such that PA images of a finite-size detector were deblurred [16]. In addition, deep learning was introduced into the OR-PAM by Davoudi; a U-net was utilized so that the OR-PAM could eliminate artifacts and improve the signal-to-noise ratio [17]. However, these methods require significant amount of data for training. Moreover, in some cases, the training set must be significantly large to deal with the high dimensionality.

In this study, the MEMS-based acquisition system was combined with the machine learning method. Furthermore, a simplified model, denominated using a spatial weight matrix (SWM), was constructed for the image reconstruction of MOR-PAM. An SWM has a hierarchical structure that contains a denoise layer, a registration layer, and a deconvolution layer. In addition, the algorithm was modularized to enable the reuse of functional modules. The performance of the SWM was evaluated quantitatively based on mutual information (MI) and the peak signal-to-noise-ratio (PSNR).

## Methods

### MEMS-based OR-PAM

**Fig. 1:**
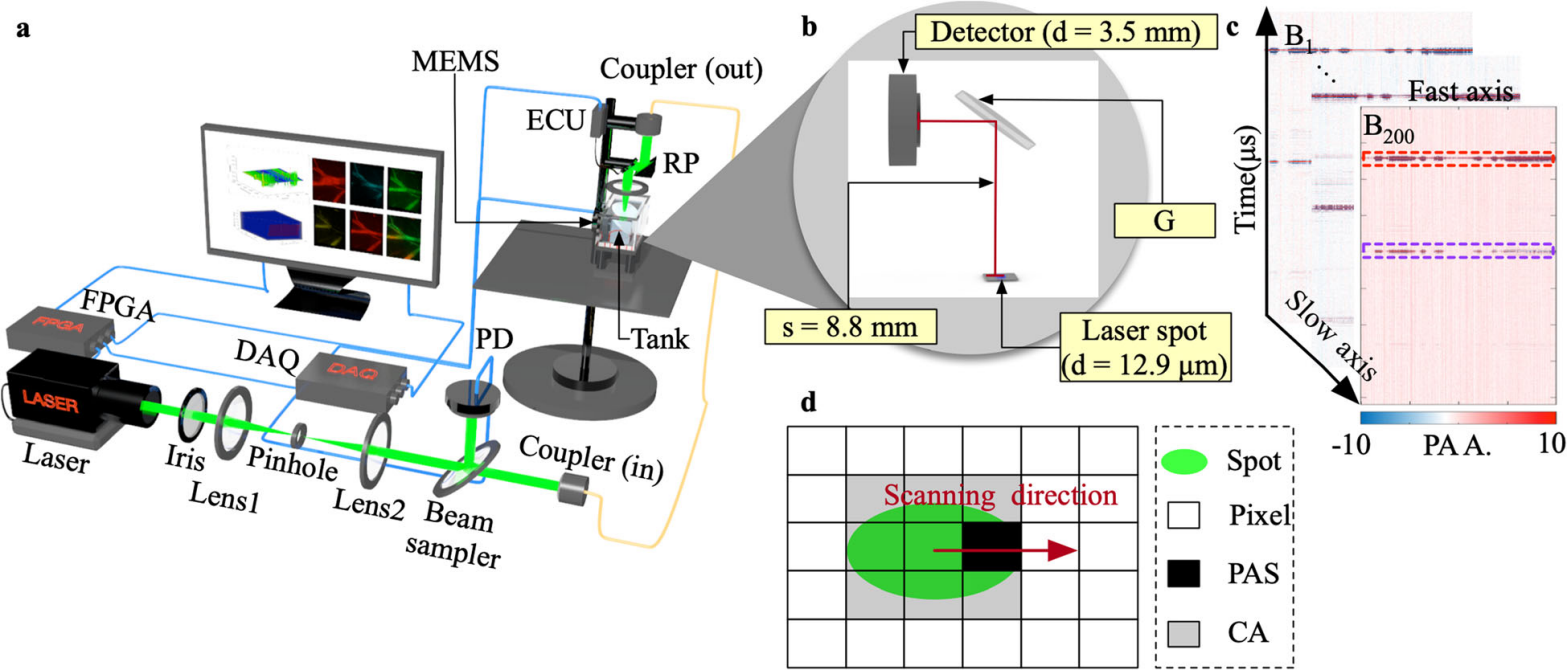
An overview of MEMS-based photoacoustic microscopy system. **a**: Schematic of the MOR-PAM; **b**: Zoomed-in views of the ultrasonic sensor; **c**: B-Scan images associated with the acquired PA matrix, the PA signals in the red dashed line, and PA signals in the purple dashed line are utilized for reconstruction; **d**: Projection of the laser spot in MAP image. FPGA: Field programmable gate array; DAQ: Data acquisition unit; MEMS: Micro-electromechanical systems; PD: Photodiode; ECU: Electronic control unit; G: Glass; RP: Reflection prism; s: Scoustic path; PAS: Photoacoustic source; CA: The estimated convolution area; PA A.: Photoacoustic amplitude.

The imaging system used in this study was the same as that used in previous studies, as shown in Figs. 1a and b [10]. The figure shows a system with the optical components annotated along the light path. The laser (532 nm, DTL-314QT, Russia) is utilized as the source for obtaining high-absorption-contrast signals, and the path is designed with a symmetric light path structure. The scanning velocity and FOV are controlled using a two-axis thermoelectric MEMS; four anchor poles of the MEMS are adjusted using a voltage array in a field-programmable gate array [18, 19]. Figure 1c shows the B-scan images of the MOR-PAM, which show significant differences in the baseline offset. In addition, it is advantageous to utilize multiple detected PA signals for an image reconstruction when the estimated noise is stable.

The resolution of the MEMS-based PAM corresponds to the step size of the MEMS mirror, which is set to 4 μm on the fast axis ($\overrightarrow{x}$) and 6 μm on the slow axis ($\overrightarrow{y}$). However, the 12.5 μm airy disk was larger than the step size; thus, the resolution was lower than expected. Moreover, the thermal effects of a repeated pulse lead to changes in the temperature-dependent Grüneisen parameter, which affects the point spread function (PSF) of the system [20]. The projection of the laser spot onto the pixel plane is shown in Fig. 1d, where a pixel is approximated as a square with a 1:1 aspect ratio. The entire signal acquisition process was reviewed, and the imaging model was divided into three layers for simplification:

1 $$ {I}^{\prime }=\left(\left(\left(\left(I+{N}_1\right)\otimes {K}_{laser}\right){M}_{map}\right)+{N}_2\right)\otimes {K}_{electric} $$

where *I′* is the detected 3D data matrix; *I* is a 3D image matrix; ⊗ is a convolution operator; *M*_*map*_ refers to the location mapping matrix, which is equal to the velocity integration of the MEMS mirror and is reversible in this case. In addition, *K*_*laser*_ refers to the laser-induced spatially dependent convolution kernel corresponding to the spot size, signal reception time, and thermal effects. Moreover, *K*_*electric*_ is the convolution kernel corresponding to an ultrasonic sensor that is spatially dependent [21, 22]. *N*_1_ is the noise produced by the interaction between laser and matter, and *N*_2_ is the noise caused by ultrasonic sensors. The inversion of the above equation is as follows:

2 $$ \hat{I}=\left(\left(\left({I}^{\prime }+N\right)\otimes {K}_{denoise}\right){M}_{map}^{-1}\right)\oslash {K}_{laser} $$

where ⊘ is a 3D deconvolution operator. In addition, *N* is the sum of the noise in this system. Because the electric impulse and frequency response are not considered in this study, ⊘*K*_*electric*_ is replaced by ⊗*K*_*denoise*_ for feature extraction. Thus, the process inversion is performed in the order of a suitable performance filtering, registration mapping, and laser-generated 3D deconvolution operation.

### Feature points extraction

The nonlinear response of the MEMS mirror introduces a pairwise deviation between a pixel grid and spot location grid if they are placed in the same coordinate frame. The corner points on the grid resolution chart are considered feature points. Our first attempt was to directly extract the feature points using adaptive non-maximal suppression and the Harris Stephen algorithm (H&SA). However, the results lag the expected outcome [23]. Thus, a pre-processing is necessary for labeling the region of interest (ROI) with ‘1’ and the other regions with ‘0’, and the ROI from the input images can be distinguished. Hence, the image cannot be segmented using a one-step method, and the objective function based on the ordinal-valued Markov random field (OV-MRF) is utilized to label the ROI through multiple iterations for a feature extraction. Here, OV-MRF is selected to perform the multi-threshold segmentation, which is suitable for the segmentation of complex references. First, the signal baseline offset of each A-line is corrected, and the baseline offset estimation in this program is presented as follows:

3 $$ \hat{f}\left({A}_h\right)=\frac{1}{\left({n}_{nd}-{n}_{st}\right)}\overset{n_{nd}}{\sum \limits_{h={n}_{st}}}{A}_h-0, $$

where $\hat{f}\left({A}_h\right)$ is the estimation of the baseline offset included in the offset matrix; *A*_*h*_ is the amplitude along the A-line with index h; *n*_*nd*_ and *n*_*st*_ are the endpoint and starting point indexes for a baseline offset estimation in a specified A-line. The offset matrix is utilized to pull the baseline back to zero.

Through repeated sampling, the 3 × 3 window was used for a local PSNR estimation. Thus, the adaptive Wiener filter with the same size was constructed by adjusting the weights using this estimated PSNR.

After the baseline offset correction and denoising, the bi-Gaussian probability distribution with a mean value pair (*μ*_1_, *μ*_2_), which is a type of universal approximator, is chosen as the prior probability distribution to iteratively divide the threshold. Note that a pre-division is recommended prior to the iteration. The objective equation for point *P* with coordinates (*m*, *n*) is as follows:

4 $$ E\left({I}_{m,n}\right)=\underset{I_{m,n},w}{argmin}w\mid {I}_{m,n}-{I}_{m,n}^{\prime}\mid +\overset{1}{\sum \limits_{\varDelta m=-1}}\overset{1}{\sum \limits_{\varDelta n=-1}}\left(\frac{1-w}{8}\right)\mid {I}_{m,n}-{I}_{m+\varDelta m,n+\varDelta n}^{\prime}\mid $$

*Δm*, *Δn* ≠ 0; *Δm*, *Δn* ∈ *Z*^∗^. Here, ${I}_{m,n}^{\prime }$ refers to the pixel value at (*m*, *n*), *I*_*m*, *n*_ refers to the pixel value at (*m*, *n*) in a binary image, and *w* is the switch function (*w* = 0, 1). Thus, the intensity of pixel *P*(*m*, *n*) is determined by the tradeoff between adjacent pixels and itself. In addition, the termination condition is set to break the loop if the condition is satisfied while the maximum iteration number is set, and a simulated annealing based on a stochastic gradient descent is proposed to improve the performance. After being labeled using the OV-MRF, the feature points are identified through the H&SA, and the adjacent corners are suppressed. In addition, the intersection of the filtering results of the Sobel edge detection with a fast and slow axis is computed to verify the results from the H&SA.

### Strategy for 3D deconvolution

The difference between a photoacoustic wave and an ultrasound wave is based on whether the emitting is active or passive. Therefore, it is easier for ultrasound waves than for an omnidirectional PA wave to focus within a smaller angle range [24]. According to the single PA source excitation formula:

5 $$ {p}_0\left(\overrightarrow{r}\right)=C\left(\theta, \phi \right)\cdotp \frac{\partial p\left({d}_{PO}\right)}{\partial {d}_{PO}}\cdotp \frac{cos\theta}{d_{PO}} $$

where ${d}_{PO}=\mid \overrightarrow{r}-{\overrightarrow{r}}_0\mid$ is the distance between the source and sensor, *C*(*θ*, *ϕ*) refers to a coefficient that varies with *θ* and *ϕ*, *p*_0_ refers to the initial pressure of the source (t = 0), and ${p}_{d_{PO}}$ is the measured data, where point P is the moving point in the ROI and point O is the fixed point for the position of the sensor. Here, $\theta =<\overrightarrow{x},\overrightarrow{k}>$, *θ* refers to the angle between the wave vector and fast axis, and $\phi =<\overrightarrow{y},\overrightarrow{k}>$, *ϕ* refers to the angle between the wave vector and slow axis. In this code, *d*_*PO*_ is refined by a linear interpolation in the initial grid.

The surface area of the unfocused ultrasonic sensor is measured at π·3.50^2^ mm, as shown in Fig. 1b, and the distance between the sensor and object is within the interval 8.80 mm. Therefore, a large error is caused if the sensor area is considered as a point on the wavefront. In this case, an acoustic source described as a voxel that has been detected over a period of time leads to an overlap on the A-lines, and the earlier detected voxel affects the intensity of the voxel detected after it. A modified delay-and-sum method (DAS) is employed to alleviate the interference in 3D microscopy [25–27]. The acoustic waves are considered as non-coherent. Owing to the superposition principle, the reconstructing formula is improved as follows:

6 $$ {p}_0\left(\overrightarrow{r}\right)=\overset{M}{\sum \limits_{m\in \varPhi }}{W}_m\left(\theta, \phi \right)\cdotp \frac{\partial p\left({d}_{P_mO}\right)}{\partial {d}_{P_mO}}\cdotp \frac{\mathit{\cos}{\theta}_m}{d_{P_mO}} $$

Here, *Φ* refers to the wavefront, the center of which is located on the A-line, and the points on the wavefront *Φ* reach this center at the same time; in addition, *W*_*m*_(*θ*, *ϕ*) refers to the weight of the correlated pixel in the local subset.

The detection area is 1.20 mm × 0.80 mm; therefore, the first detected voxel will influence the voxel microscopy until the end of this process. The distances of the PA sources (Fig. 1d) are too close to be distinguished in the lateral direction by the sensor. The deconvolution is estimated by attaching the additional weight matrix, which is determined by calculating the average value of the photoacoustic signal in this area during the time reversal process. Thus, the method of local weighting is helpful in reducing the symmetric artifacts and the generalization errors in this complex task [28, 29]. In this case, it will enhance an already strong source and weaken a weak source in the fixed deconvolution area; in addition, the edges of the ROI are enlarged. To balance this enhancement and restore the edges, the filtering process is guided by the image before deconvolution [30].

Thus, a whole image with a high SNR is obtained. Instead of confronting the special deconvolution problem for every pixel in this image, a shared decoding method with a specified scope is employed for each subset, which is based on the systematic features. To reduce time complexity, the iteration operation is replaced by a matrix multiplication during the programming.

### SWM

**Fig. 2:**
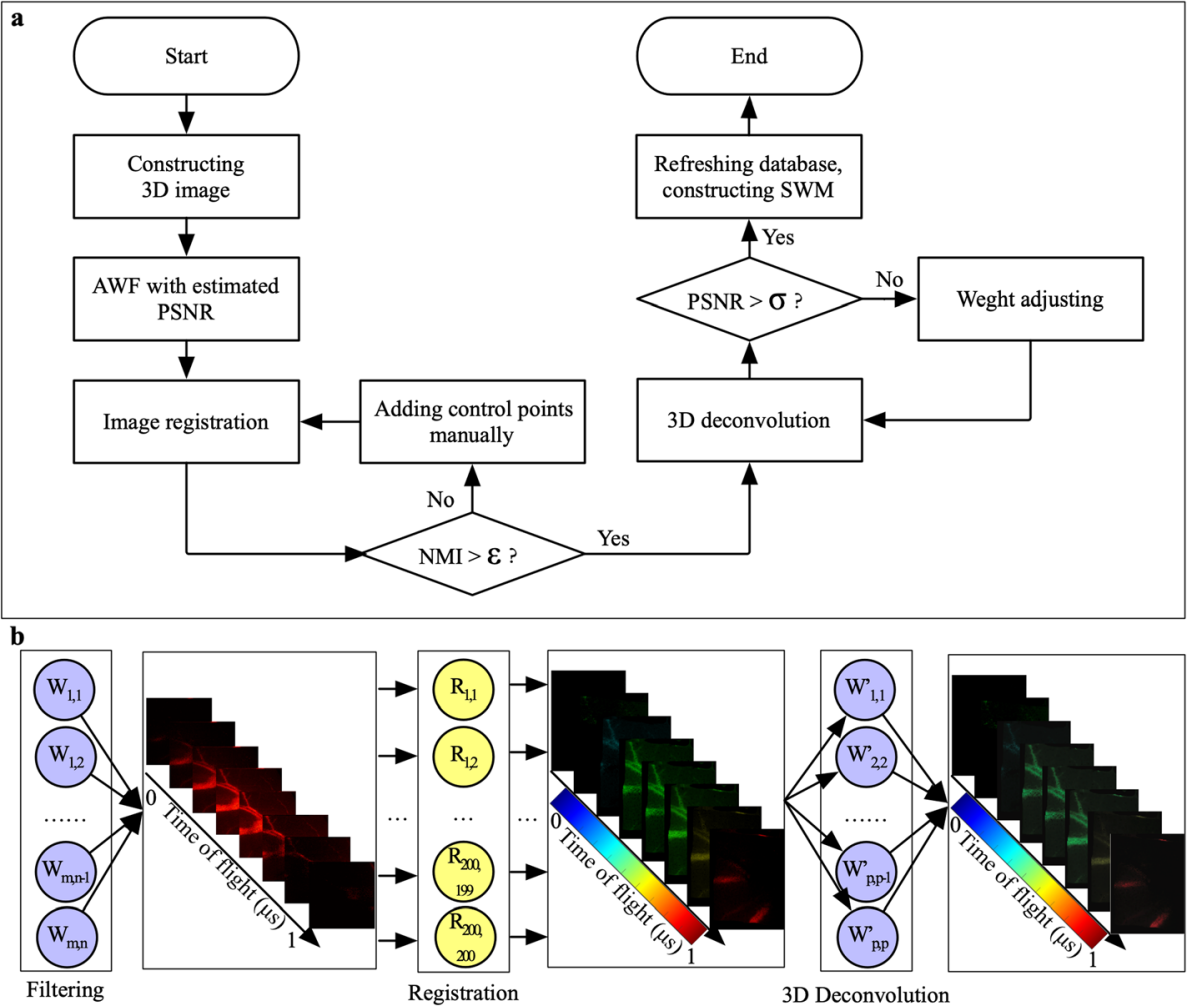
Overall design of SWM. **a**: Flowchart of constructing SWM; **b**: Image processing flow through an SWM hierarchical structure. AWF: Adaptive Wiener filtering; NMI: Normalized mutual information; PSNR: Peak signal-noise-ratio; *W*_*i*, *j*_: The element in an adaptive Wiener filter; *R*_*i*, *j*_: The element in a registration matrix; ${W}_{i,j}^{\prime }$: The element in a deconvolution kernel

Although the lateral resolution of the MOR-PAM benefits from a tightly focused optical beam and high-performance ultrasonic sensor, this is difficult to realize through engineering [31, 32]. An SWM is proposed to correct the distortion and perform a deconvolution. A flowchart of constructing the SWM is shown in Fig. 2a, and the processing flow with the SWM is shown in Fig. 2b. The image of each layer has a size of 200 × 200, and the time-of-flight represents the time when the image is detected. The noise reduction, registration of 2D images, and 3D deconvolution were employed to reduce the signal superposition. In addition, the 3D image is obtained through an interpolation. The construction of the SWM corresponds to a reversal of the wave propagation, as indicated in Eq. 2.

**Fig. 3:**
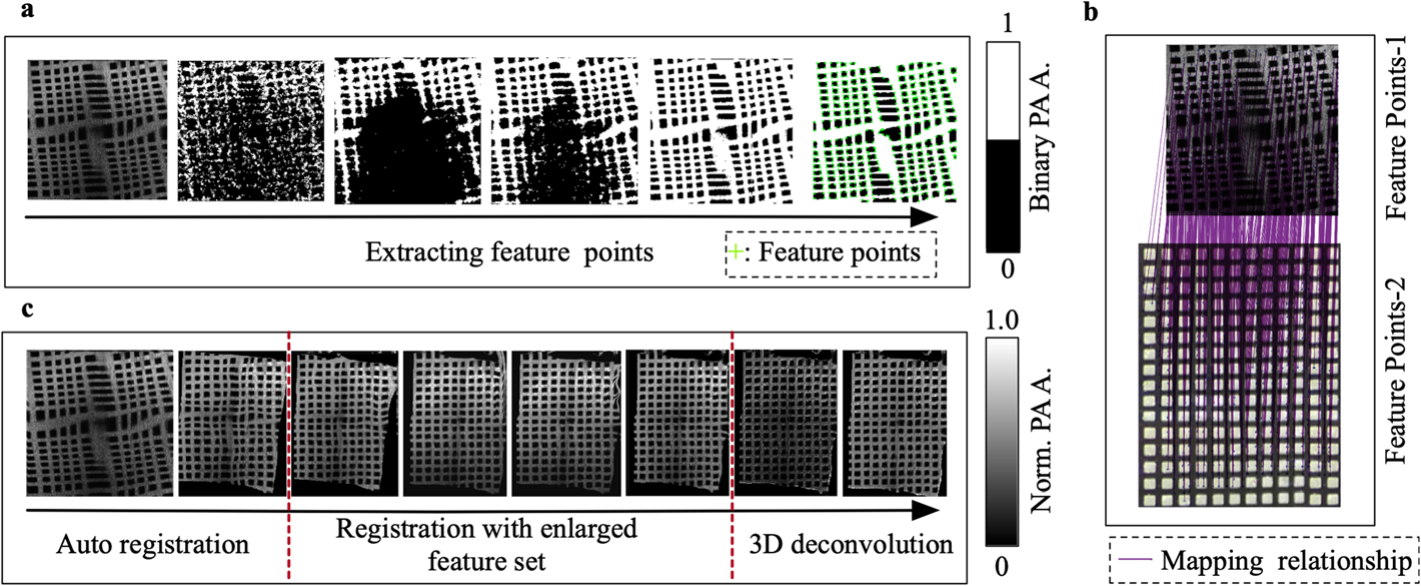
Stage results of SWM construction with a resolution chart. **a**: The process of extracting feature points by utilizing OV-MRF and H&SA; **b**: The mapping relationship between the feature points-1 (the feature points in the input image) and the feature points-2 (the feature points in reference image); **c**: The process of image registration based on the feature point pairs; stage results after the first red dashed line are obtained by adding manually selected feature points for registration, and stage results after the second red dashed line are 3D deconvolution results. Norm.: Normalized; PA A.: Photoacoustic amplitude

The mapping relationship between the input and output is stored in the SWM, which has a hierarchical structure with three layers. The first layer corresponds to the denoising filter, which is used to reduce the system noise *N*. The adaptive Wiener filter based on the statistics of the A-line energy is utilized. In this study, the filter size is 3 × 3. The second layer is the registration matrix, which refers to the location mapping between distorted images and reference images. As described in subsection Feature points extraction, by extracting the feature points from the reference image and the distorted image, the feature point pairs are grouped into a sparse set. Figure 3a shows the process of extracting the feature points using OV-MRF and H&SA. The feature points are labeled with a green ‘+’ in the last image of the sequence. Thus, the geometric transformation is inferred from this sparse correspondence. In this study, the MATLAB method fitgeotrans is used to estimate a geometric transformation. As shown in Fig. 3b, the relationship between feature points in the input image and feature points in the reference image is plotted with purple lines. Figure 3c shows the process of an auto registration and a registration with enlarged feature points. Moreover, the deconvolution and image-guided results are displayed at the end of the image array. The last layer is the 3D deconvolution layer obtained by employing a modified DAS in the deconvolution area, as described in subsection Strategy for a 3D deconvolution.

A mapping relationship corresponding to different spot sizes, ultrasonic sensors, and the scanning speed of the MEMS mirror is constructed in advance. Consequently, the image reconstruction of MOR-PAM is considered as a knowledge-based geometric transformation [33]. The 3D image is reconstructed using the specified SWM along the wavefront propagation direction.

### Evaluation metrics

Normalized mutual information (NMI) is a method used to measure the correlation between two sample sets based on the joint entropy, namely, the transmission of information is acquired from an observation of sample A to the knowledge of sample B [34, 35]. The principle of MI is outlined as follows:

7 $$ I\left(A,B\right)=H(A)+H(B)-H\left(A;B\right) $$

Here, *H(A)* and *H(B)* are the entropy of images A and B, respectively, and *H(A; B)* refers to the joint entropy of A and B. NMI is acquired by normalizing the calculated MI *I(A, B)*. To calculate the MI in a computer, images A and B are converted into a grayscale image with specified levels. Subsequently, considering image A as the column and image B as the row, the joint histogram is substituted into the entropy calculation formula to obtain *H(A; B)*. The equation for computing entropy is given as follows:

8 $$ {I}_{NMI}(A)=\frac{-1}{C}\overset{M}{\sum \limits_{m=1}}\overset{N}{\sum \limits_{n=1}}P\left(A\left(m,n\right)\right)\cdotp lo{g}_2\left(P\left(A\left(m,n\right)\right)\right) $$

Here, *C* refers to the normalization parameter. In addition, the PSNR, as a full reference image quality evaluation index, is introduced to evaluate the 3D deconvolution.

## Results

Considering that the COVID-19 pandemic is still a global problem, the experimental data for validation of this SWM are transplanted from our database, which includes the data from the PAL equipped with electrothermal MEMS [10] and the data from a photoacoustic pen equipped with electrostatic MEMS [9].

### Environment and kits

The program was operated on a MacBook with 8 GB RAM and 1.53 GB VRAM, using a CPU with a processing speed of 2.90 GHz, which can satisfy the requirements of non-parallel computation. In addition, all images were processed on MATLAB R2020a for academic use with some convenient kits downloaded from GitHub. The algorithm design was accelerated using the unified modeling language (UML), and simulations were carried out on MATLAB.

### Experiments on test images

The test images for the SWM validation include an image of the resolution chart with a ring texture and images of vessels in a rat abdomen. The complete process is divided into the following stages:

The first stage was the construction of the SWM. Considering the thermal effects, an enlarged airy disk was considered as the top area of the 3D deconvolution area, and the height of the deconvolution area was estimated as 1.05 mm. The feature points of the resolution chart were extracted for estimating the registration mapping. The number of point-pairs for an electrothermal MEMS registration was 783, which included some manually added points; in addition, the number of point-pairs for registering the electrostatic MEMS was 78. After registration, the modified DAS was employed for a deconvolution, and the result of 3D deconvolution was considered as the guided filter, which is filtered by the result of registration. In this experiment, the neighborhood size of the guide filter was 3 × 3.

An electrothermal MEMS was then employed in the raster scanning. A continuous Dirac comb controlled the angular speed of the micro-scanner with a frequency of 10 kHz, based on which the time interval of two adjacent points was estimated at approximately 125 μm. The scanning area was set to 1.20 mm × 0.80 mm. In addition, the warm-up time required was considered for ensuring the stable performance of the electrothermal MEMS.

The last stage was an image reconstruction using the SWM to correct the distortion and improve the SNR of the images.

**Fig. 4:**
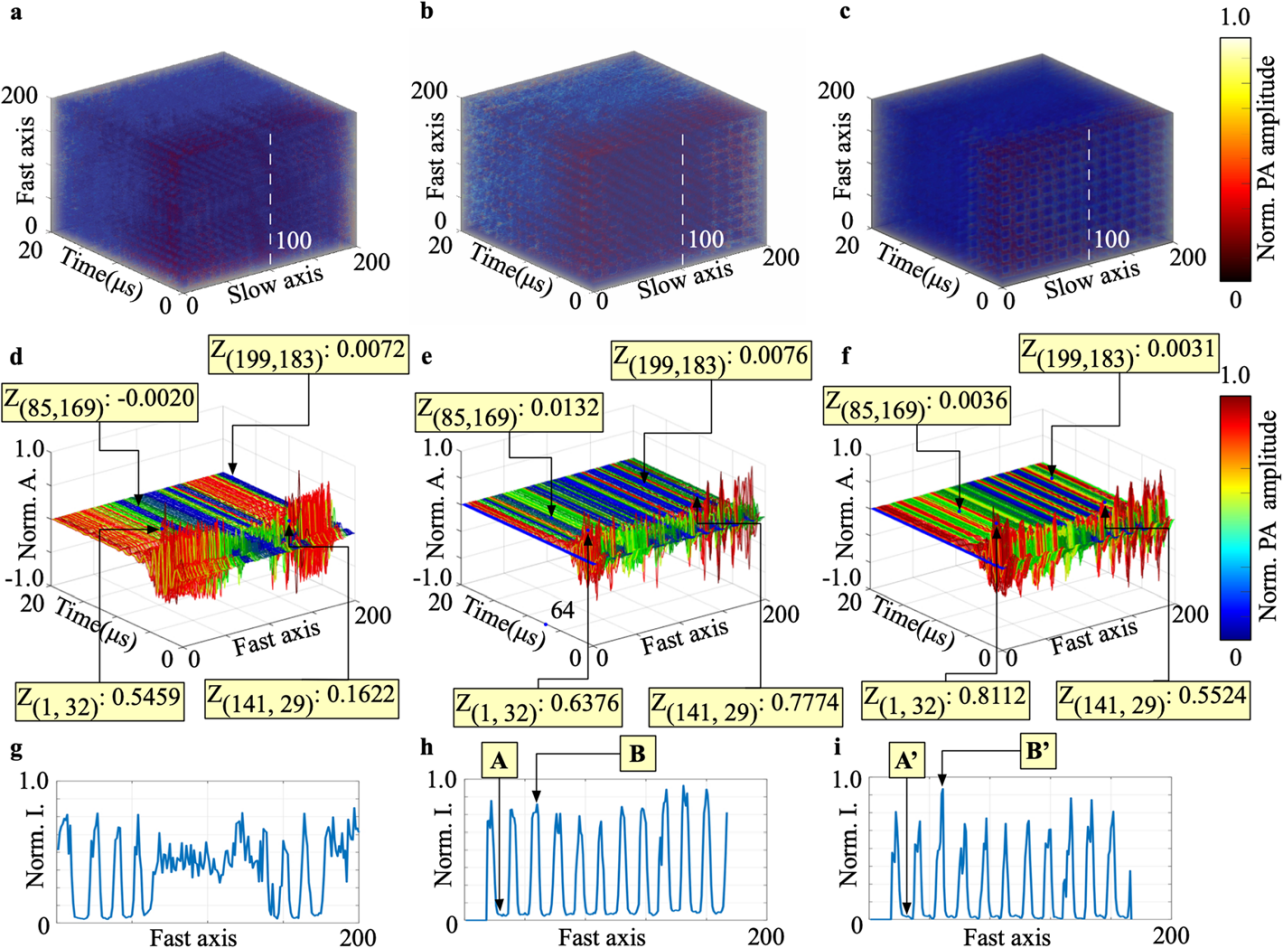
Analysis of using SWM to process a resolution chart. **a**: Original 3D image; **b**: The result of denoising and registration of Fig. **a**; **c**: 3D deconvolution result of Fig. **b**; **d**-**f**: A-lines stack of white dashed line in Figs. **a**-**c**, respectively; **g**-**i**: The profiles of MAP images of Figs. **a**-**c** marked with the white dashed line. Norm.: Normalized; A., Amplitude; I.: Intensity

The obtained B-scan images are stacked, and 200 slice images from 691 to 890 were extracted as a ROI. The input 3D image is shown in Fig. 4a. The 3D image (Fig. 4b) was generated after registration, as shown in Fig. 4a. Using Fig. 4b as the input image, the 3D image (Fig. 4c) was generated after a 3D deconvolution. Both images are displayed in sequence for comparison. Some differences between Fig. 4a and b can clearly be seen; in addition, the signal value is increased after a 3D deconvolution, as shown in Fig. 4c. The images in Figs. 4d-f correspond to the slices marked with the white dashed line in Figs. 4a-c, respectively. The image in Fig. 4e is more orderly than that in Fig. 4d; meanwhile, Fig. 4f shows an improved SNR compared with Fig. 4e. The profiles of line 93 in the MAP images of Figs. 4a-c are then obtained and displayed in Figs. 4g-i, respectively. The value at point A’ related to the noise is smaller than that of point A; moreover, point B, which is the local maximum value, is further increased to point B′.

**Fig. 5:**
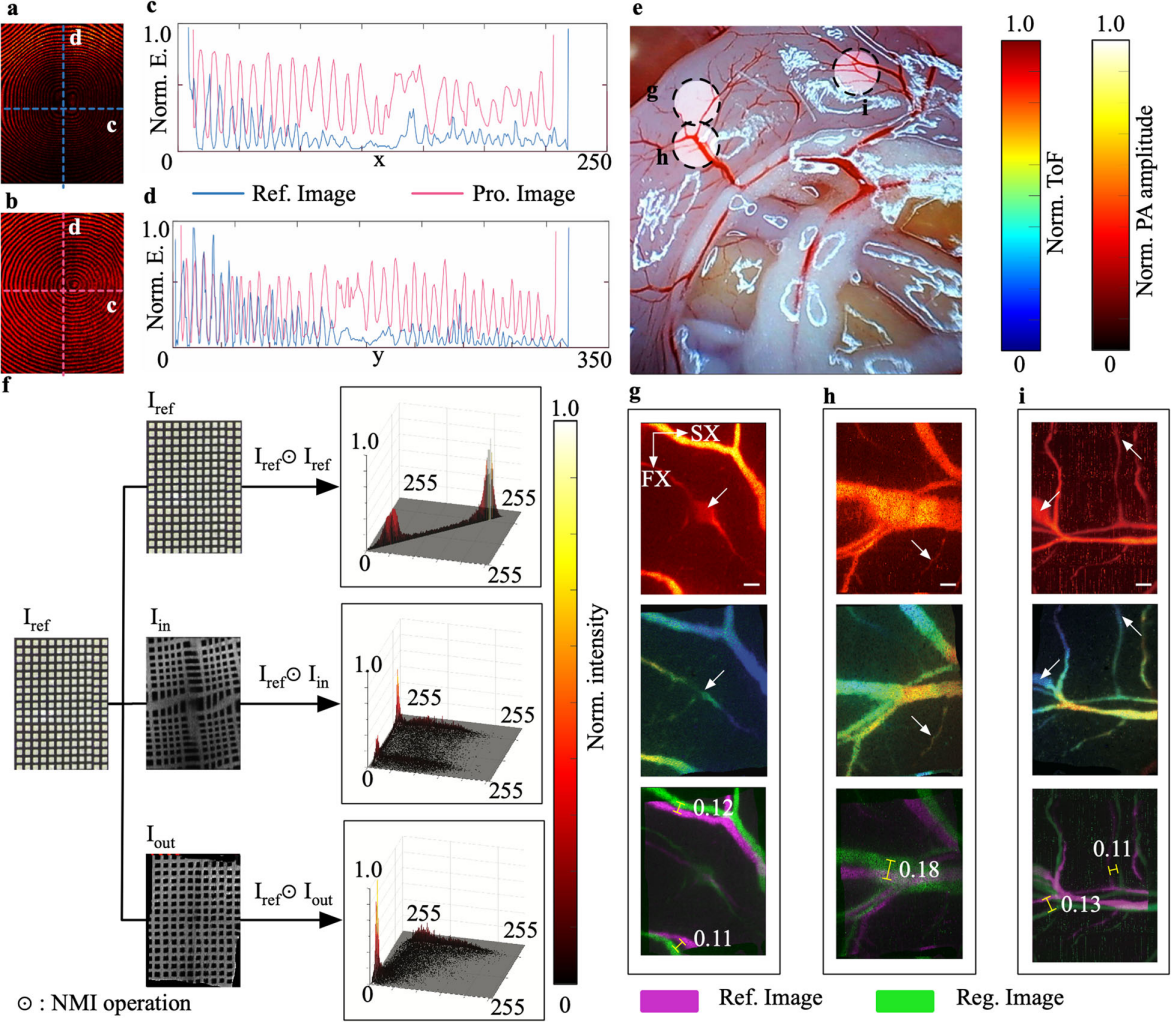
Experimental results of image reconstruction with SWM. **a**: Original MAP image of a ring texture resolution chart; **b**: The processed MAP image of Fig. **a**; **c**: Horizontal profile along colored dashed-lines in a and b rendered with different colors; **d**: Vertical profile along colored dashed-lines in Figs. a and b; **e**: Image of vessels in the abdomen of a laboratory rat; **f**: Joint histogram comparison of different images; **g**-**i**: The above images of g-i are distorted images; the middle images are the corrected depth-coded images (processed images); the lower images of **g**-**i** are superimposed images between the original images and the processed images; white-arrows and rulers are utilized to mark obvious correction. Norm.: Normalized; ToF: Time of flight; Pro.: Processed; Ref.: Reference; Reg.: Registered; units: μm

MOR-PAM images of a resolution chart with a ring texture and rat vessels were reconstructed to verify the effectiveness of the SWM (Fig. 5). A MAP image of the resolution chart with a ring texture is shown in Fig. 5a, and a processed image of the resolution chart is displayed in Fig. 5b. The profiles of the red- and blue-dashed lines in Figs. 5a and b are plotted in Figs. 5c and d. In this experiment, black cardboard was used as the scattered sample to measure this difference for compensation. In addition, the MOR-PAM images are displayed in Fig. 5g-i, and the image of the vessels in the rat abdomen is shown as a reference (Fig. 5e).

By employing MI, the effectiveness of the SWM was quantitatively measured. Meanwhile, it was utilized as an evaluation metric during an iterative registration. The *ε* in the flowchart (Fig. 2a) was selected to be 0.48. The magnitude of *ε* is reasonable for judging the effectiveness of the correction because the mean value of the PA images is lower than that of the images in visible light. The joint histogram was drawn in MATLAB for comparison, as shown in Fig. 5f. The self-joint histogram of the image of the resolution chart is a bi-Gaussian profile along the diagonal, whereas the raised points on the joint histogram between the distorted image and the chart image is unevenly distributed on the left half-plane. After processing with the SWM, the distribution is pulled back diagonally and distributed in the lower triangle because the intensity of the PA is lower than that of its image. The calculation results of the NMI are as follows: the joint entropy *H (I*_*out*_*, I*_*ref*_*)* was 13.35 in the 256-bin-sized histogram when the entropy *H (I*_*in*_*)* was equal to 6.99. In addition, the NMI *I*_*NMI*_ *(I*_*out*_*, I*_*ref*_*)* was 70.33-times larger than *I*_*NMI*_ *(I*_*in*_*, I*_*ref*_*)* in a binary histogram.

### Experiments on depth-coded images

MOR-PAM was utilized to detect the images in the deep layers, which could not be observed directly. The data from the photoacoustic pen equipped with electrostatic MEMS in our database were chosen to verify the performance of the SWM in 3D images. As shown in supplementary, the oral blood vessels of humans were rendered in color along the axis direction with the colormap jet in MATLAB. Three slices with equal spacing along the axis direction were selected as the MAP plane for comparison.

## Discussion

In this study, the image processing of MOR-PAM was divided into three stages corresponding to three layers. First, a hierarchical processing was used to simplify the physical model such that it can be easily coded and extended in the future. In addition, the SWM was constructed using a modified DAS in the 3D deconvolution area instead of a 2D deconvolution, which is reasonable for MOR-PAM. Moreover, the grid resolution chart, which turns a nonlinear distortion correction into a knowledge-based registration, was verified experimentally to facilitate distortion correction. In the application of a convolutional neural network, the SWM can also find its own position. For example, a requirement of fully connected layers is a consistent input image size generated through the SWM; in addition, high-quality images (reference images) can be estimated with the help of the SWM for supervised training.

The SWM can be used for MOR-PAM reconstruction, although some imperfections still exist. For example, the distortion of the electrothermal MEMS is highly susceptible to the temperature; thus, the temperature of the environment and the untimely heat dissipation effect on the scanning speed as a thermal drift. Therefore, when using an electrothermal MEMS for detection, the thermal drift may produce additional distortion that is difficult to estimate in advance. This limitation can be addressed by constructing an adaptive SWM, which can identify and correct the distortion during sampling. As another approach, we can construct sufficient number of SWMs prior to sampling, and the most suitable SWM will reconstruct the distorted image.

In addition, we did not consider the PSF associated with the ultrasonic sensor, and the area of 3D deconvolution was based on an empirical assumption. A suitable method is to use a generative adversarial network to estimate the size of the convolution kernel.

Our next study will include an image reconstruction using a combination of a preset SWM and dynamically adjusted weights, as well as a real-time correction system, to compensate for the unstable velocity of the MEMS.

## Conclusions

An SWM, which is a practical tool used to estimate the invariable information of a system, is a predetermined matrix that allows a specified system to reconstruct images. Similar to other traditional machine learning methods, an SWM uses a hierarchical structure to simplify the complex physical process.

First, the adaptive Wiener filter with a size of 3 × 3 is constructed for image denoising. For the process of registration, a grid resolution chart is employed to estimate this deviation for correcting the nonlinear distortion. During this process, an OV-MRF is used to label the ROI to help the H&SA in the corner point extraction. In addition, the intersection of the filtering results of the Sobel edge detection along the fast and slow axes is computed to verify the results from the H&SA. Here, it is necessary to select some feature points manually to guarantee a high-performance registration. Therefore, the extracted feature points are employed to estimate the geometric transformation from a distorted image to a ground-true image.

In addition, a 3D deconvolution is applied to deal with overlapped signals, which leads to an edge degradation. To balance this effect of the deconvolution, we use a guided filter to restore the boundary while retaining the deconvolution results.

This distortion is caused by the system characteristics. Therefore, we can construct a different SWM according to different conditions (such as different MEMS mirror, scanning velocity, spot size, and ambient temperature) and apply this mapping relationship to reconstruct the images detected under the same condition. The contributions of this work include the following:

A processed SWM is constructed in advance for the reconstruction of MOR-PAM images. In the image processing using an SWM, some details of the image processing modules, such as a 3D deconvolution and baseline offset correction, are coded for the reconstruction; these modules are useful in other types of image reconstruction. In addition, an SWM is expected to be used for a real-time distortion correction by monitoring the offset dynamically.


**Additional file 1.** The comparison of human oral microvascular images processed by SWM with minimum filter.

## Data Availability

All the functional modules will be shared on GitHub.

## Abbreviations

SWM: Spatial weight matrix
MEMS: Micro-electromechanical system
OR-PAM: Optical-resolution photoacoustic microscopy
PAM: Photoacoustic microscopy
MOR-PAM: MEMS-based optical-resolution photoacoustic microscopy
FOV: Field of view
MAP: Maximum amplitude projection
PSNR: Peak signal-to-noise-ratio
ROI: Region of interest
OV-MRF: Ordinal-valued Markov random field
NMI: Normalized mutual information
MI: Mutual information
UML: Unified modeling language
