# Correction to: Spatial weight matrix in dimensionality reduction reconstruction for micro-electromechanical system-based photoacoustic microscopy

**DOI:** 10.1186/s42492-020-00066-6

**Published:** 2020-12-21

**Authors:** Yuanzheng Ma, Chang Lu, Kedi Xiong, Wuyu Zhang, Sihua Yang

**Affiliations:** 1 MOE Key Laboratory of Laser Life Science & Institute of Laser Life Science, College of Biophotonics, South China Normal University, Guangzhou, 510631 China; 2 Guangdong Provincial Key Laboratory of Laser Life Science, College of Biophotonics, South China Normal University, Guangzhou, 510631 China

**Correction to: Vis Comput Ind Biomed Art 3, 22 (2020)**

**https://doi.org/10.1186/s42492-020-00058-6**

Following publication of the original article [1], the keywords are missing in the article.

Keywords: Photoacoustic microscopy, Spatial weight matrix, Dimensionality reduction, Distortion correction, Mutual information

The original article has been updated.
